# Better together: antiobesity medications and bariatric surgery for the management of obesity

**DOI:** 10.1093/bjs/znae294

**Published:** 2024-11-29

**Authors:** Dimitri J Pournaras, Ildiko Lingvay, Priya Sumithran

**Affiliations:** Department of Bariatric and Metabolic Surgery, North Bristol NHS Trust, Bristol, UK; Division of Endocrinology, Department of Internal Medicine and Peter O’Donnel Jr School of Public Health, University of Texas Southwestern Medical Center, Dallas, Texas, USA; Department of Surgery, School of Translational Medicine, Monash University, Melbourne, Victoria, Australia; Department of Endocrinology and Diabetes, Alfred Health, Melbourne, Victoria, Australia

As evidence for the efficacy and safety of the new generation of obesity medications accumulates, it is timely to reconsider the approach to obesity treatment. Although bariatric surgery remains the most effective and durable intervention, new medications are rapidly gaining popularity because they are perceived as a more approachable and reversible option. Long-term studies comparing the effectiveness, safety, and cost-effectiveness of bariatric surgery with those of new antiobesity medications are lacking, and would be informative. However, rather than viewing these highly effective and complementary treatment approaches as inevitable competitors, learning in whom, when, and how to combine them could be even more valuable and relevant to patient care.

Obesity is a highly stigmatized chronic, relapsing disease. To treat it optimally, lessons from other chronic conditions could be adopted. As in cancer care, combining obesity treatment modalities such as pharmacotherapy, endoscopic therapies, use of devices, and bariatric surgery may well be the way forward, either before surgery (neoadjuvant) or after operation (adjuvant or rescue)^1^. How to accomplish this is underinvestigated, as highlighted by the systematic review^2^ of the available evidence demonstrating the research gaps and paucity of high-quality RCTs. Resolving this will require evidence to fill the gaps in understanding of the fundamental principles of obesity care that have been exposed by the emergence of more effective treatment approaches.

One such example is: what is the appropriate treatment target? Is it loss of a certain percentage of bodyweight (say 15%)—a relative number linked to an arbitrary (and variable) starting point?^3^ Is it reversal of obesity-associated health and functional impairments? But would that be enough to prevent their subsequent progression? Is it a bodyweight (BMI) or anthropometric measure of adiposity within the range deemed ‘normal’ or ‘low risk’? But how would this risk be defined, given that estimates of population-level risk are not suitable for individuals?

An international consensus^4^ by a multidisciplinary team of experts in obesity management has provided some useful and authoritative recommendations, and a practical blueprint for obesity care to temporarily navigate this uncertainty. However, it is clear that high-quality research is imperative to inform evidence-based care. Clinical trials to establish the long-term safety, efficacy, and cost-effectiveness of multimodal strategies are urgently needed, as well as studies that inform optimal patient selection for different treatment approaches.

Perhaps the recent successes in this field are also the biggest challenge. The growing number of promising therapies currently under investigation adds another layer of complexity in designing long-term studies that will remain relevant by the time they are completed. Although the challenges remain numerous, so do the opportunities, so we should not be deterred from tackling the right clinical questions.

We are at a seminal time in obesity care. To paraphrase Sir Winston Churchill: this is not the end. Not even the beginning of the end. But perhaps this is the end of the beginning. The future looks very bright, but we need the right studies to inform our approach and best treatment algorithms to optimize the care of people whose health is impaired by this highly prevalent disease.
